# Role of Body Mass Index and gestational weight gain on preterm birth and adverse perinatal outcomes

**DOI:** 10.1038/s41598-019-49704-x

**Published:** 2019-09-11

**Authors:** Fabia Pigatti Silva, Renato T. Souza, Jose G. Cecatti, Renato Passini, Ricardo P. Tedesco, Giuliane J. Lajos, Marcelo L. Nomura, Patricia M. Rehder, Tabata Z. Dias, Paulo F. Oliveira, Cleide M. Silva, Brazilian Multicenter Study on Preterm Birth (EMIP) study group, Maria L. Costa, Rodolfo C. Pacagnella, Samira M. Haddad, Vilma Zotarelli, Lucio T. Gurgel, Nelson L. Maia Filho, Jacinta P. Mathias, Sergio T. Marba, Ruth Guinsburg, Francisco E. Martinez, Silvana M. Quintana, Patrícia P. S. Melli, Francisco E. Feitosa, George N. Chaves, Ana M. Porto, Isabela C. Coutinho, Antonio C. Barbosa Lima, Elias F. Melo, Débora F. Leite, Melania M. Amorim, Adriana S. O. Melo, Fabiana O. Melo, Marília G. Martins, Marinea V. Nunes, Cláudio S. Paiva, Moises D. Lima, Djacyr M. Freire, Edson G. Tristão, Denis J. Nascimento, Carlos A. Menezes, Marcelo Aquino, Janete Vettorazzi, Cintia E. Senger, Augusta M. B. Assumpção, Marcela A. F. Guedes, Maria E. L. Moreira, Vera T. Borges, Eduardo Souza, Ana C. P. Zamarian, Fátima A. Lotufo, Kaliane Uzilin, Elvira A. Zanette, Carla B. Andreucci, Tenilson A. Oliveira, Laércio R. Oliveira, Marcos A. N. Santos, Nelson Sass, Mirian R. F. Silveira, Pedro R. Coutinho, Luciana Siqueira

**Affiliations:** 1 Department of Obstetrics and Gynecology, School of Medical Sciences, University of Campinas (UNICAMP), Campinas, SP Brazil; 2 Department of Obstetrics and Gynecology, Jundiaí Medical School, Jundiaí, SP Brazil; 3 Unit of Statistics, School of Medical Sciences, University of Campinas (UNICAMP), Campinas, Brazil; 4 Department of Pediatrics, School of Medical Sciences, University of Campinas (UNICAMP), Campinas, SP Brazil; 5 Division of Neonatal Medicine, Escola Paulista de Medicina (EPM), Universidade Federal de São Paulo (UNIFESP), São Paulo, SP Brazil; 6 Faculty of Medicine Ribeirão Preto, Universidade de São Paulo, São Paulo, Brazil; 7 Maternity School, Assis Chateaubriand, Fortaleza, Ceará Brazil; 8 Instituto de Medicina Integral Prof. Fernando Figueira, Recife, Pernambuco Brazil; 9 Department of Mother and Child Health, School of Medicine, Federal University of Pernambuco, Recife, Brazil; 10 Instituto de Saúde Elpidio de Almeida, Campina Grande, Paraíba Brazil; 11 Departmanet of Gynecology and Obstetrics, Federal University of Maranhão (UFMA), São Luiz, MA Brazil; 12 Federal University of Paraíba, João Pessoa, Paraíba Brazil; 13 Federal University of Paraná, Curitiba, Paraná, Brazil; 14 Federal University of Bahia, Salvador, Bahia Brazil; 15 Federal University of Rio Grande do Sul, Porto Alegre, RS Brazil; 16 Instituto Fernandes Figueira, Rio de Janeiro, RJ Brazil; 17 Department of Gynecology and Obstetrics, São Paulo State University, Botucatu, São Paulo Brazil; 18 Department of Obstetrics, Paulista School of Medicine, Federal University of São Paulo (EPM-UNIFESP), São Paulo, SP Brazil; 19 Santa Casa de Limeira Hospital, Limeira, SP Brazil; 20 Santa Casa de São Carlos Hospital, São Carlos, SP Brazil; 21 Maternity Casa Maternal Leonor Mendes de Barros, São Paulo, SP Brazil; 22 Maternity Escola de Vila Nova Cachoeirinha, São Paulo, SP Brazil; 23 Hospital Estadual de Sumaré, Sumaré, SP Brazil

**Keywords:** Weight management, Weight management, Risk factors, Risk factors

## Abstract

The association of body mass index (BMI) and gestational weight gain (GWG) with preterm birth (PTB) remains controversial in the literature. To evaluate different maternal BMI and GWG categories, according to the initial BMI, in relation to different PTB subtypes and perinatal outcomes, we conducted a secondary analysis of a multicentre cross-sectional study, along with a nested case-control study including PTB from 20 centers in Brazil. Pre-pregnancy underweight was associated with a lower risk of provider-initiated PTB, while overweight and obesity were associated with a higher risk of provider-initiated PTB and a lower risk of spontaneous preterm birth. Insufficient gestational weight gain was associated with a higher prevalence of spontaneous PTB and preterm premature rupture of membranes. Excessive GWG correlated with a higher prevalence of provider-initiated PTB or preterm premature rupture of membranes. Irrespective of the initial BMI, the greater the rate of GWG, the higher the predicted probability of all PTB subtypes, except for spontaneous PTB in underweight women and those with normal BMI. On multivariate analysis, the initial BMI was shown to be the only factor associated with pi-PTB. Briefly, further studies evaluating the risk for PTB should consider that GWG may have a different role depending on the initial BMI and PTB subtype.

## Introduction

Preterm birth (PTB) is an increasing health concern, as well as a major cause of neonatal mortality and long-term morbidity worldwide^1^. To develop effective strategies to reduce preterm birth, it is important to assess the causes and risk factors for the condition. Body mass index (BMI) before and during early pregnancy and gestational weight gain (GWG) during pregnancy have already been associated with preterm birth. However, their exact role in determining preterm birth remains to be determined^2^.

Obesity and overweight are recognized as growing global health problems^3^. The prevalence of overweight adult women increased from 29.8% in 1980 to 38% in 2013 worldwide, especially in middle-income countries^4^. Major adverse health outcomes are related to obesity in pregnant women, including gestational diabetes, pregnancy-induced hypertension, preeclampsia, postpartum hemorrhage, and caesarean delivery^5^. Pregnancy-related complications are known to contribute to medically indicated PTB and occur more commonly in overweight and obese women^2,6^. In contrast, low BMI in early pregnancy has been consistently reported as a risk factor for preterm birth, particularly spontaneous preterm birth (sPTB), when compared to any other weight status^7–9^.

Considerably fewer studies have evaluated the association between gestational weight gain and preterm birth. Contradictory results have occurred in many cases, and most studies were conducted in high-income countries with different contexts, such as racial, cultural, and socioeconomic factors, compared to low and middle-income countries^7–9^. In addition, these studies have generally failed to distinguish between different preterm birth subtypes (sPTB, PROM-PTB and pi-PTB) and the rate of gestational weight gain, limiting their ability to delineate the dose-response relationship between gestational weight gain and preterm birth subtype^10–12^. Therefore, BMI and gestational weight gain in early pregnancy should still be evaluated, in association with preterm births and perinatal outcomes, regarding the rate of gestational weight gain as a modifying factor for adverse maternal and perinatal outcomes in the developing world^13^.

A retrospective cohort study with almost 9 thousand women delivering singleton babies from 2006 to 2009 in Lima, Peru, showed an independent association between the rate of gestational weight gain and preterm birth (especially sPTB), which varies depending on pre-pregnancy BMI. This association was protective in underweight women. Nevertheless, in overweight women and those with a normal BMI, both very low rates and very high rates of gestational weight gain were associated with an increased rate of preterm births. These results are important for public health and highlight the need for further studies to expand our knowledge on the determinants of preterm birth^10^.

This study is part of the Brazilian Multicenter Study on Preterm Birth (EMIP)^14^, one of the most comprehensive epidemiological studies on preterm birth in Brazil, conducted in 20 referral obstetrical facilities from different geographical regions of the country. The purpose of this analysis is to evaluate the association of pre-pregnancy or early pregnancy BMI and gestational weight gain with the risk of preterm births, along with their subtypes. Secondly, we aim to assess the impact of gestational weight gain and pre/early pregnancy BMI on the severity of adverse perinatal outcomes among preterm births.

## Results

**Figure 1:**
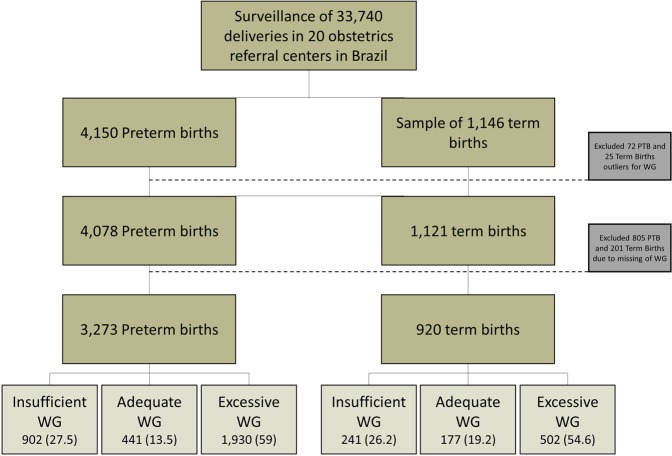
Flow chart of study participants according to adequacy of weight gain (WG).

Of the total of 33,740 births surveyed by the EMIP study, preterm births numbered 4,150 and 1,146 births were selected to build the control group of term births (Fig. 1). After excluding outlier data and considering all preterm and term births, 4,506 (85%) had information about early/pre-pregnancy BMI and 4,193 (79.2%) had complete information for calculation of gestational weight gain (Fig. 1). Although the majority of women had normal pre-pregnancy BMI (56.1%), approximately 85% were considered to have inadequate gestational weight gain, either insufficient or excessive (data not shown). Additionally, more than one-third of women were overweight or obese at the beginning of pregnancy (35.4%).

Table 1 shows the risk estimates for preterm birth according to maternal early/pre-pregnancy BMI and adequacy of GWG during pregnancy. Overweight and obesity were associated with a higher risk of pi-PTB, despite a lower risk of sPTB. Underweight was associated with a 40% lower risk for pi-PTB. Insufficient rate of GWG during pregnancy, regardless of the initial BMI, was associated with an increased risk for sPTB (1.7-fold) and PROM-PTB (1.5-fold). Women with excess rate of GWG were more likely to have PROM-PTB (1.4-fold) and pi-PTB (2 -fold).

**Table 1:** Risk estimates of different subtypes of preterm births according to maternal initial body mass index (BMI) and adequacy of weight gain.

	sPTB	OR* (95% CI)	PROM-PTB	OR* (95% CI)	pi-PTB	OR* (95% CI)	Term birth
**Initial Body Mass Index** ^**a**^							
Underweight	144 (11.5)	1.26 [0.93–1.70]	100 (9.8)	1.16 [0.84–1.60]	56 (4.5)	**0.62 [0.43–0.89]**	82 (8.3)
Normal	782 (62.6)	1	577 (56.6)	1	604 (48.2)	1	565 (57.4)
Overweight	226 (18.1)	**0.74 [0.59–0.92]**	210 (20.6)	0.92 [0.73–1.16]	338 (27.0)	**1.43 [1.16–1.77]**	209 (21.2)
Obese	98 (7.8)	**0.54 [0.40–0.72]**	132 (12.9)	0.99 [0.75–1.31]	254 (20.3)	**1.76 [1.37–2.26]**	129 (13.1)
**Adequacy of Weight Gain** ^**b**^							
Insufficient	401(34.5)	**1.76 [1.34–2.31]**	271 (28.6)	**1.54 [1.15–2.06]**	230 (19.8)	1.28 [0.95–1.71]	241 (26.2)
Adequate	172 (14.8)	1	133 (14.0)	1	136 (11.7)	1	177 (19.2)
Excessive	590 (50.7)	1.19 [0.93–1.53]	543 (57.3)	**1.45[1.11–1.88]**	797 (68.5)	**2.01 [1.56–2.59]**	502 (54.6)
Total	1,470		1,173		1,435		1,121

OR*: Odds ratio adjusted for the cluster effect design in comparison to term birth group. **CI:** Confidence interval.

**sPTB:** Spontaneous preterm birth. **PROM-PTB:** preterm premature rupture of membranes. **pi-PTB:** provider-initiated preterm birth.

Missing information for: a) 693 b) 1006 cases.

Values in **bold** mean they are statistically significant.

**Figure 2:**
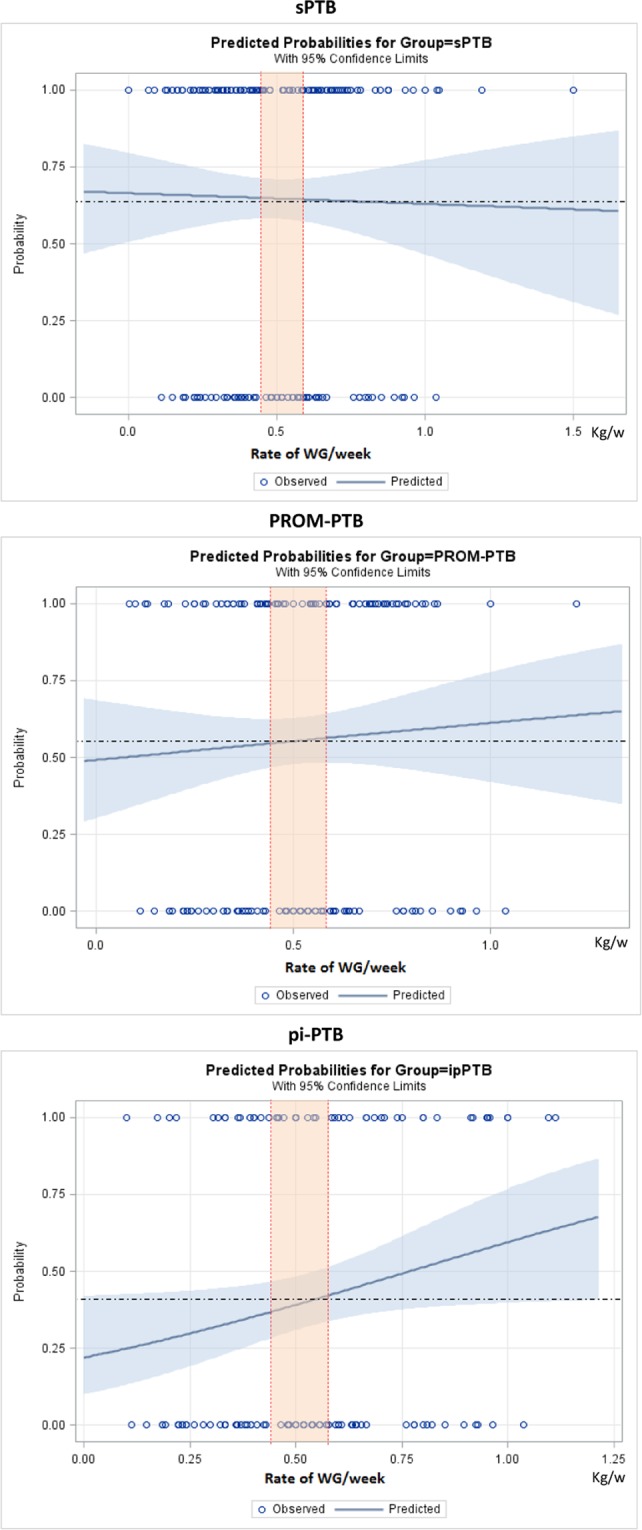
Probability of different types of preterm births for underweight women according to rate of weight gain.

**Figure 3:**
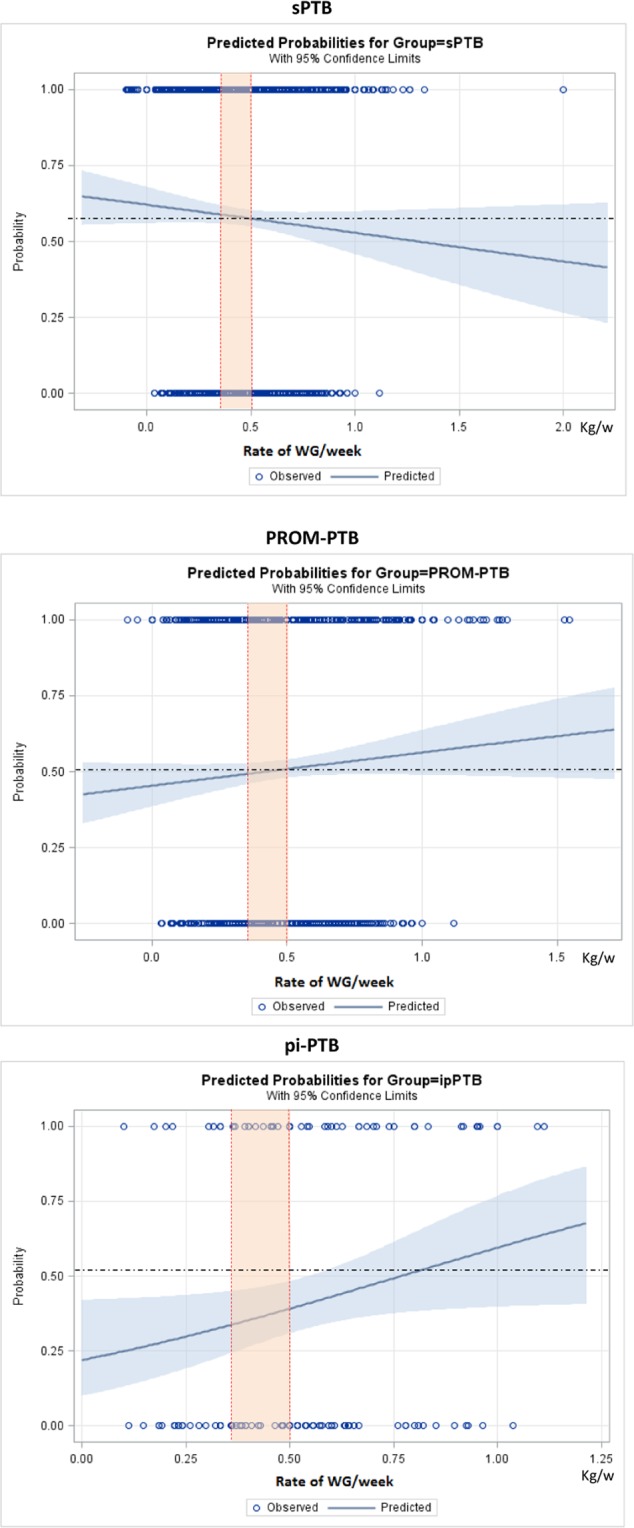
Probability of different types of preterm births for women with normal weight according to rate of weight gain.

**Figure 4:**
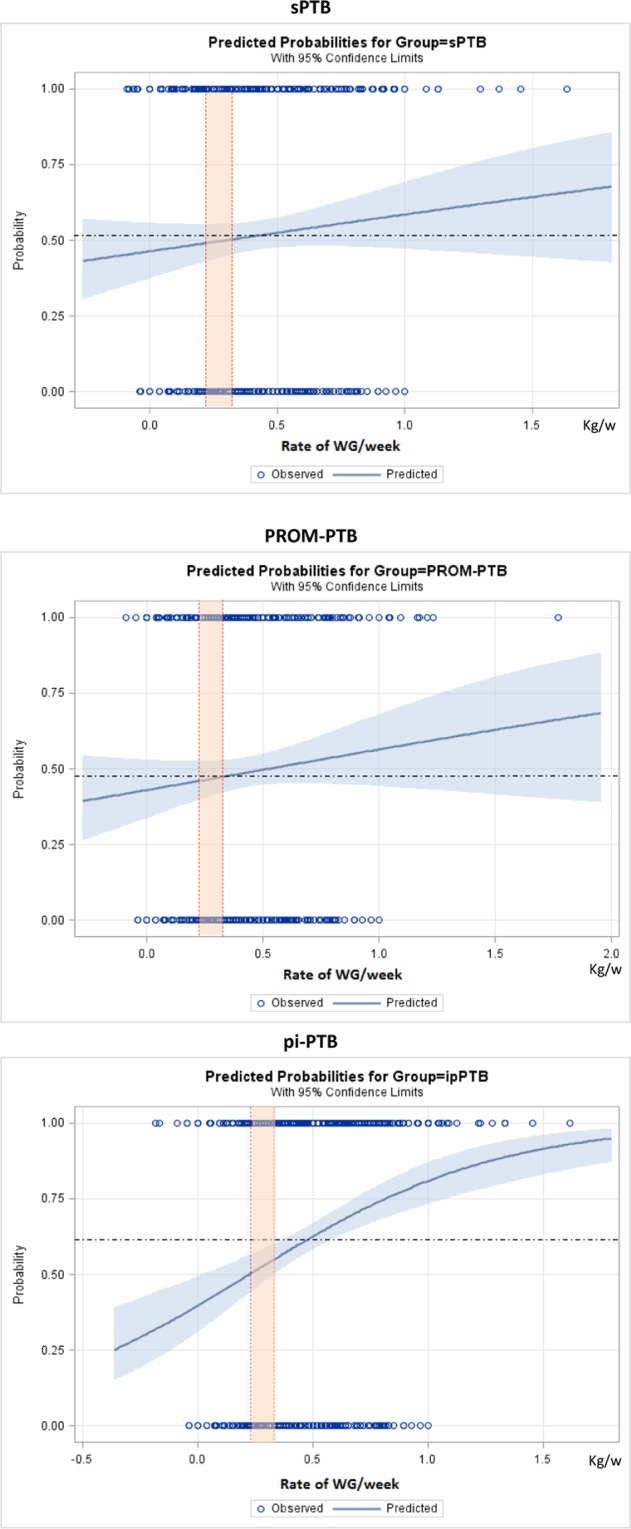
Probability of different types of preterm births for overweight women according to rate of weight gain.

**Figure 5:**
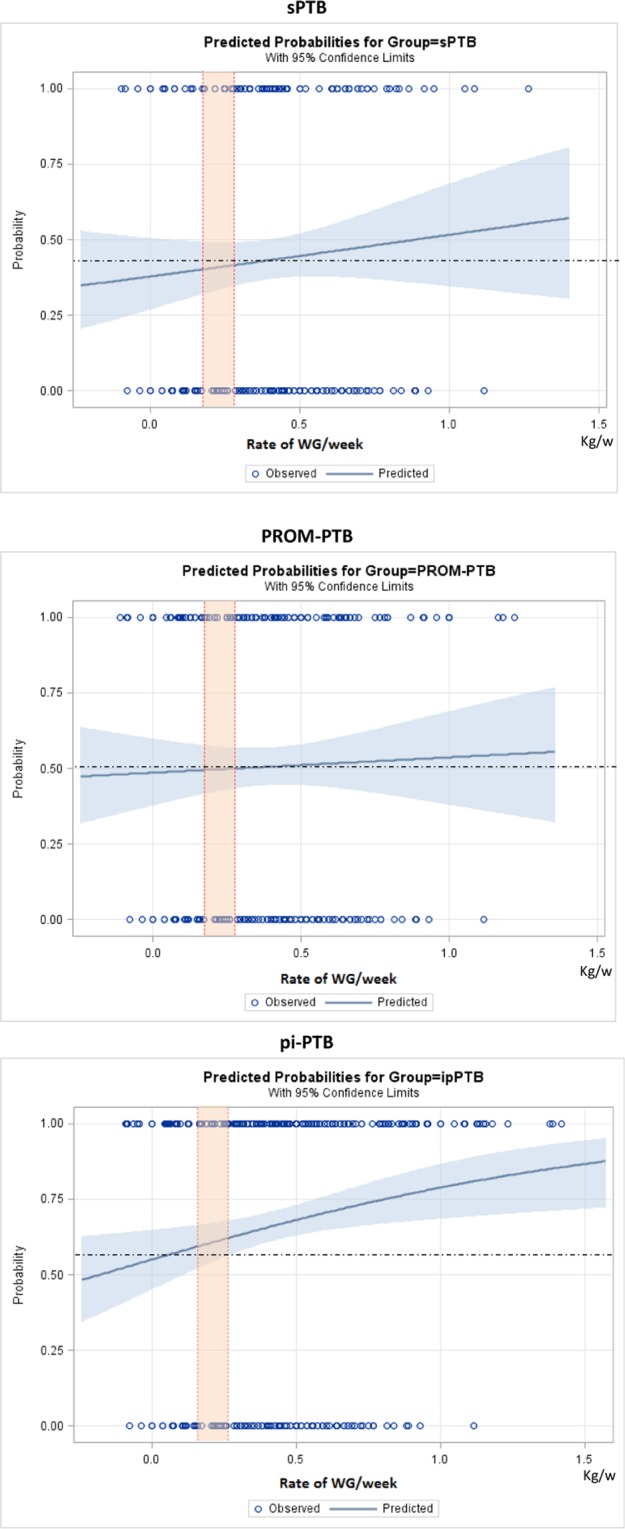
Probability of different types of preterm birth for obese women according to rate of weight gain.

Figures 2–5 show the predicted probabilities of preterm birth subtypes for underweight women, those with normal BMI, overweight women and obese women, respectively, according to the rate of GWG (per week). In overweight and obese women, the higher the rate of gestational weight gain, the higher the predicted probability of occurring all subtypes of preterm birth. In underweight women or in those with normal BMI, the trend towards increased probability according to higher rates of GWG only remains for PROM-PTB and pi-PTB. Nonetheless, the probability of spontaneous preterm birth in underweight women remains bordered on stable irrespective of the rate of GWG, while it decreases the higher the rate of GWG in women with normal BMI.

Women with insufficient rates of GWG had a proportionally higher prevalence of preterm infants below 28 weeks and between 28 and 33 weeks of gestation (Table 2). Newborns of women with insufficient rates of GWG had a higher proportion of NICU admission.

**Table 2:** Perinatal outcomes according to adequacy of weight gain during pregnancy in preterm births.

Perinatal outcomes	Adequacy of Weight Gain			
	Insufficient	Adequate	Excessive	p-value
**Gestational age [n = 3273]**				**<0**.**0001**
<28 weeks	65 (7.2)	23 (5.2)	134 (3.2)	
28–33 weeks	261 (28.9)	108 (24.5)	562 (23.9)	
34–36 weeks	576 (63.9)	310 (70.3)	1234 (72.9)	
Apgar score <7 at 5 minutes [n = 3234]	96 (10.8)	30 (6.9)	179 (9.4)	0.0511
NICU admission[n = 3060]	653 (64.3)	602 (57.2)	598 (60.3)	**0**.**0045**
Fetal death [n = 3273]	41 (6.4)	11 (2.9)	71 (2.1)	0.1714
Neonatal death before discharge [n = 3127]	71 (8.3)	25 (5.9)	139 (7.5)	0.2996
Any adverse perinatal outcome (APO)^*^ [n = 3273]	522 (57.9)	233 (52.9)	1133 (58.7)	0.0786

^*^Any adverse perinatal outcome (APO): Apgar score <7 at 5 minutes **or** NICU admission **or** neonatal death before discharge.

P-values in **bold** mean they are statistically significant.

Multivariate analyses showed that fetal malformation, history of vaginal bleeding during pregnancy, maternal morbidity and multiple pregnancies were independently associated with any adverse perinatal outcome (APO) in women with preterm births (Table 3). Adequacy of GWG was not shown to be independently associated with any APO, while initial BMI was weakly associated with pi-PTB.

**Table 3:** Variables independently associated with any adverse perinatal outcome (APO) in women with all types of preterm births: stepwise multiple analyses by non-conditional logistic regression.

Any preterm birth [n = 3040]	OR_adj_	95% CI	p-value
Fetal malformation	6.73	4.85–9.34	<0.0001
Vaginal bleeding	1.42	1.19–1.71	0.0001
Maternal morbidity	1.31	1.12–1.55	0.0010
Multiple pregnancy	1.42	1.09–1.84	0.0079
**Spontaneous preterm birth [n = 1093]**			
Fetal malformation	8.35	4.75–14.67	<0.0001
Multiple pregnancy	1.88	1.28–2.77	0.0013
Vaginal bleeding	1.61	1.20–2.17	0.0016
Number of C-sections	0.77	0.61–0.97	0.0281
**Preterm birth due to PROM [n = 898]**			
Fetal malformation	7.72	4.13–14.46	<0.0001
Vaginal bleeding	1.65	1.16–2.28	0.0047
Number of C-sections	1.47	1.11–1.95	0.0068
**Provider-initiated preterm birth [n = 1049]**			
Fetal malformation	5.01	2.87–8.74	<0.0001
Initial BMI	0.97	0.95–0.99	0.0178

**OR**_**adj**_: Odds ratio adjusted for cluster effect design and for all predictors in this final model; **CI:** confidence interval of OR; **p:** p-value. **Predictors entering the model:** maternal age, parity, number of previous vaginal births, number of previous cesarean sections, number of abortions (nulliparous: 0/≥1: 1); schooling (≥12:0, <12:1), adequacy of weight gain during pregnancy (Adequate:0/Insufficient or Excessive:1); initial BMI (kg/m2); maternal morbidity* (no: 0/yes: 1); vaginal bleeding (yes: 1/ o: 0) multiple pregnancy (yes: 1/no: 0); fetal malformation (yes: 1/no: 0).

*Defined as having any of the following: anemia, chronic hypertension, pre-pregnancy diabetes, gestational diabetes, gestational hypertension, preeclampsia/eclampsia/HELLP, hypo/hyperthyroidism, HIV, cardiac disease, renal disease, lung diseases, epilepsy, systemic lupus erythematosus or thrombophilia/thrombosis.

## Discussion

The EMIP study was a comprehensive survey on preterm birth in Brazil. During the study, it was confirmed that the prevalence of 6.5% was underestimated according to updated official data of the Brazilian Government (around 10% in 2011)^15^. Therefore, the surveillance of less than 37,000 deliveries was sufficient to achieve the number of participants for each group. Maternal characteristics of EMIP study have been already published elsewhere^14,16^. Nevertheless, we highlight that more than one-third of participating women were overweight or obese at the beginning of pregnancy. High estimated rates of overweight or obese Brazilian women aged 25–34 years justify the rising concern about endemic obesity and overweight, especially in middle- and high-income countries. Traditionally, early BMI has been used as a proxy for nutritional status at the beginning of pregnancy^17^, and it has been largely studied as a risk factor for preterm birth^2,18,19^. Although it is conventional, early BMI is an unmodifiable marker for the index pregnancy. Therefore, the current analyses included the evaluation of gestational weight gain during pregnancy, since it could represent a more dynamic and modifiable nutritional status during pregnancy.

There are conflicting results in the literature regarding the risk of preterm birth, and early maternal BMI^2,18,19^. In general, underweight has been related to a higher risk of sPTB and obesity related to PROM and pi-PTB. However, many studies do not consider the preterm subtypes separately in the analyses, and this is the main limitation to systematic reviews, as well as a uniform categorization of BMI. According to a systematic review of maternal BMI and risk for PTB, despite the existence of 39 studies on this topic published over 40 years, the lack of BMI standardization places a limit on analyses and weakens the results and evidence^2^. The calculation of gestational weight gain is also another potential limitation in observational studies, mainly in retrospective cohorts. Many studies calculate total gestational weight gain, which complicates a comparison between a preterm and term delivery, and does not take into account the expected lower weight gain in gestations of shorter lengths. Two recent systematic reviews addressed the risks for adverse neonatal outcomes according to IOM categories for gestational weight gain. Goldstein *et al*.^20^ and Kominiarek *et al*.^21^ showed that total weight gain below the recommended values were associated with a higher risk of preterm birth with OR 1.70 (95% CI [1.32–2.20]) and OR 1.47 (95% CI [1.31–1.64]), respectively.

In contrast to previously described IOM guidelines^22^, the results of the EMIP study suggest that abnormal BMI and inadequate gestational weight gain may have different effects on the risk of preterm birth subtypes. In our study, overweight and particularly obese women were associated with a higher risk for pi-PTB, despite a lower risk for sPTB. Obesity and overweight are established risk factors for metabolic disorders such as gestational diabetes, hypertension, preeclampsia, polyhydramnios and others, which are associated with maternal complications and consequently with a higher risk for pi-PTB^2,16,23^. Observational studies suggest that myometrial function is affected in obese women due to an abnormal response to oxytocin. Oxytocin receptors are decreased in myometrial biopsies in obese women at term^24–27^. This alteration is linked to higher rates of postdate pregnancies and slower labour progression in obese women, in comparison to non-obese women. Furthermore, there is low evidence in the hypothesis about the effects of endogenous oxytocin on food intake and body weight. Higher levels of endogenous oxytocin and/or overexpression of oxytocin receptors may reduce food intake and increase energy expenditure, reducing body weight^28^. Taking that into account, these women would have a higher risk for prematurity, despite a protective mechanism for obesity.

On the other hand, a population-based cohort study of almost 1.6 million singleton deliveries in Sweden from 1992 through 2010 showed an increased risk for sPTB, especially extremely preterm infants, in overweight and obese pregnant women^29^. Obesity is characterized by inflammatory up-regulation, associated with proinflammatories cytokines and adipokines and alterations of the hypothalamic-pituitary-adrenal axis, which is responsible for releasing corticotrophin-releasing hormone. In high values, it is known as a risk factor for premature rupture of membranes, preterm labour, eclampsia and pregnancy-induced hypertension^29–31^. This conflicting evidence indicates that multiple underlying mechanisms may play a role in PTB risk in women with different BMI.

According to our analyses, the effects of gestational weight gain on PTB risk were dependent on the initial BMI and preterm subtype. In general, excessive GWG was associated with a higher probability of preterm birth, particularly pi-PTB in overweight and obese women. Carnero and colleagues conducted a retrospective cohort study in Peru. Those authors found that overweight women and those with a normal BMI have a “U-shaped” curve of association, where both low and high RWG were associated with an increased risk for preterm birth, particularly pi-PTB^10^. The U-shaped form of association is in agreement with the literature, which shows that extreme rates of GWG are important risk factors for PTB^22,32–34^. Therefore, pregnant women should be in the middle of this curve to minimize risk. Although we did not observe such an association in our study, our findings demonstrate that the rate of GWG is associated with PTB risk depending on the initial BMI.

In our analyses, insufficient gestational weight gain during pregnancy was related to more severe prematurity and a higher admission to NICU. Insufficient GWG is associated with adverse perinatal outcomes such as small for gestational age and spontaneous preterm birth, but it seems to be dependent on the initial BMI category^35^. Spontaneous preterm birth was associated with insufficient GWG, but it is usually related to a lower prevalence of very and extreme prematurity than pi-PTB^36^. Therefore, the higher prevalence of sPTB in women with an insufficient GWG does not explain the higher proportion of very and extreme prematurity. A separate analysis of insufficient GWG considering different initial BMIs might be useful to determine the underlying reasons for this association.

Although our analyses were performed according to international recommendations for the rate of GWG, BMI categories and preterm subtypes, we identified some potential limitations. To accomplish this analysis, the formula for rate of GWG assumes the same weight gain in the first trimester for all women. Although the GWG recommended by the IOM in the first trimester is the same for all women irrespective of the initial BMI, having a gestational weight gain of 0.5 kg or 2 kg, could represent a substantial difference in the rate of GWG, particularly for underweight women. In addition, we were unable to address weight loss at early pregnancy. This is another important piece of information which occurs in a proportion of women and affects the first trimester and total weight gain. Another critical point was that only 8.5% of participating women in our study were underweight. The small number of underweight women weakens the analyses, especially when divided into subgroups of PTB or categories of GWG. Furthermore, the likelihood of underweight women having excessive weight gain seems to depend on ethnicity and environmental aspects^10,35,37^. In Brazil, underweight women are more likely to have insufficient gestational weight gain and unlikely to have excessive weight gain^38^. An excessive GWG for underweight and obese women might produce different effects^38^. A recent systematic review and meta-analysis gathering information on women from USA, Europe and Asia showed that ethnicity may play a major role in the association of maternal BMI and gestational weight gain with the risk of preterm birth^39^. Gestational weight gain below IOM recommendation was associated with a higher risk for preterm birth in North American and European (OR 1.35 [95% CI 1.17–1.56]), but not in Asian pregnant women (OR 1.06 [95% CI 0.78–1.44]). Authors acknowledge that IOM guidelines may not apply to all populations, as it does for Asian pregnant women. Specific normality for BMI and weight gain, along with recommended parameters should be established for each population.

Although we used standard clinical proxies for nutritional status in pregnancy, maternal BMI and weight gain during pregnancy are related to several other aspects, including biological conditions, food intake and lifestyle habits that were not assessed. Underweight was initially thought to be exclusively related to undernutrition and obesity was related to overconsumption. However, high-calorie foods that have a poor nutritional value are now linked to obesity and low socio-economic status^40^.

It has already been reported that initial BMI and gestational weight gain during pregnancy are risk factors for neonatal adverse outcomes^41^. We carried out a logistic regression analysis to identify whether there is an association between initial BMI or GWG and any adverse perinatal outcome (APO) in preterm newborns. Nevertheless, no consistent association was observed except for known risk factors such as multiple pregnancy, fetal anomaly, maternal morbidity and vaginal bleeding.

Despite the limitations of our study, important lessons may be learned in future studies evaluating the burden of initial BMI and GWG on the risk for PTB: (1) Using the rate of gestational weight gain during pregnancy (per week) and not the total weight gain, which does not take into account the length of pregnancy; (2) Considering preterm birth subtypes separately instead of preterm birth as a unique syndrome, since the underlying conditions, motivators and outcomes are not the same; and (3) Using adequate GWG during pregnancy according to the initial BMI, since weight gain may exert different effects dependent on the initial BMI category. Findings of the EMIP study highlight the need for further studies and standardization of risk assessment of preterm birth, taking into account nutritional status.

## Material and Methods

### Study design

This is a secondary analysis of data obtained from a multicenter cross-sectional study plus a nested case-control study named EMIP investigating 20 low- and high-risk healthcare facilities from three different geographical regions of Brazil (the Northeast, Southeast and South). Methodological details of the EMIP study have been previously published elsewhere^14,42^. Briefly, data was collected from April 2011 to March 2012, using a form containing 306 variables specifically developed for this study. All women with preterm births were identified and invited to participate, including those who had had multiple pregnancies and stillbirths. The very next woman with a term birth after the preterm delivery was invited to participate in the control group, until the estimated sample size was achieved. Should there be refusal to participate, the next woman was invited. Data collection procedure included an interview with participants until discharge, and a review of maternal and newborn medical records and prenatal chart. After data collection from each individual case, the form was completed and checked, and information was included in the online database system that used a special platform for clinical studies, the OpenClinica®.

The sample size was calculated using the official prevalence rate of preterm births in Brazil in 2009, which was around 6.5% at the time of the research proposal. Considering an acceptable absolute difference of about 0.25% between the sample and prevalence of the population, as well as a type I error of 5%, 37,000 deliveries were required to obtain the sample size. For the case-control component, each group (preterm subtypes and controls) had an estimated sample size of 1,054 women. Sample size calculation was based on the primary objectives of the EMIP study as previously published. The current analysis was conducted as a function of collected data.

Full ethical approval was obtained from the National Council for Ethics in Research (CONEP) and by the Institutional Review Board of each participating center. Before enrolment, all individuals signed an Informed Consent Form. Several procedures were adopted to ensure high-quality data, including preparatory meetings for training of assistants and collaborators, detailed standard operating procedure manuals (SOP’s) developed that explain management of the questionnaire and database, monitoring site visits, sustained monitoring of data entry by the coordinating center and fast identification and correction of errors. All methods were performed in accordance with the principles of the Brazilian National Heath Council (Resolution CNS 466/12). in compliance with local/institutional guidelines and regulations in all stages of this study.

### Outcomes and variables

The main outcome for this analysis is the occurrence of preterm birth, defined as delivery that occurs below 37 weeks, due to spontaneous onset of labour (sPTB), pre-labour rupture of membranes (PROM-PTB) or one that was medically indicated due to maternal or fetal compromise or both (pi-PTB). Term birth is defined as childbirth at or after 37 weeks^14,16^. Secondary outcomes were categories of gestational age (<28 weeks, 28–33 weeks and 34–36 weeks of pregnancy)^1^, fetal death, and neonatal outcomes such as Apgar score <7 at five minutes, admission to neonatal intensive care unit (NICU), neonatal death before discharge and any adverse perinatal outcome (APO: a composite variable defined as the occurrence of any previous neonatal adverse outcome).

Maternal independent variables were: pre- or early pregnancy BMI, categorized as underweight (<18.5 kg/m^2^), normal (18.5–24.99 kg/m^2^), overweight (25–29.99 kg/m^2^) and obese (≥30 kg/m^2^)^22^. Early pregnancy BMI was calculated using the first weight recorded on the prenatal chart (up to 20 weeks of gestation) and measured height. Adequacy of gestational weight gain during pregnancy was based on the rate of gestational weight gain (GWG), calculated by the following formula: rate of GWG = (first maternal weight in pregnancy - last maternal weight)/(gestational age at delivery - 12). Adequacy of gestational weight gain was then categorized as insufficient when RWG < 0.44 kg/w for underweight, <0.35 kg/w for normal, <0.23 kg/w for overweight or <0.17 kg/w for obese; adequate when RWG 0.44–0.58 kg/w for underweight, 0.35–0.50 kg/w for normal, 0.23–0.33 kg/w for overweight or 0.17–0.27 kg/w for obese; and excessive when RWG ≥ 0.58 kg/w for underweight, ≥0.50 kg/w for normal, ≥0.33 kg/w for overweight or ≥0.27 kg/w for obese, according to pre- or early pregnancy BMI as recommended by the Institute of Medicine (IOM)^22^. IOM guidelines currently recommend the same weight gain during the first trimester irrespective of BMI categories. To categorize adequacy of estimated gestational weight gain, the upper and lower limits in the first trimester are narrower in comparison to other trimesters. Taking this into account, we subtracted 12 weeks from the gestational age, since there is almost no difference in weight gain among women of different BMI during this period. Moreover, a large part of Brazilian women initiates prenatal care after the first trimester.

### Statistical analyses

Statistical analyses were conducted to estimate the risk for all PTB subtypes, using Odds Ratio (OR) with 95% confidence intervals (CI) for BMI and adequacy of gestational weight gain categories, adjusting for cluster effect design. We estimated the probability of each preterm birth subtype, according to the initial BMI category and rate of GWG during pregnancy, using binary logit analyses optimized by Fisher´s scoring. Dotted lines in each figure of predicted probability delimited the lower and upper limits of recommended weekly rate of GWG, according to the IOM. The area in orange between the lines shows the recommended rate of GWG for each initial BMI. The upper and lower values are considered excessive and insufficient rates of GWG, respectively. The occurrence of adverse perinatal outcomes according to adequacy of gestational weight gain during pregnancy, which is controlled by gestational age, was evaluated by χ^2^ tests. Statistical significance level was set at p-value < 0.05. Stepwise multiple analysis by non-conditional logistic regression was carried out to identify factors that were independently associated with APO in women with preterm births. To estimate the likelihood of each preterm birth subtype according to early BMI, we dismissed outliers of weight gain, ignoring data on gestational weight gain above the 99^th^ percentile and below the 1^st^ percentile. Statistical analyses were performed using SAS System for Windows (Statistical Analysis System), version 9.4. SAS Institute Inc, 2002–2008, Cary, NC, USA. Institute Inc, 2002–2008, Cary, NC, USA. This manuscript follows the STROBE statement^43^.

## Data Availability

All relevant data are included in the paper, and the authors can make materials, data and associated protocols available, if requested.
